# ﻿Comparative cytogenetics among populations of two *Bothriurus* species (Scorpiones, Bothriuridae)

**DOI:** 10.3897/compcytogen.19.165160

**Published:** 2025-11-13

**Authors:** Juliana F. de Lima, Marielle C. Schneider, Leonardo S. Carvalho, Ricardo Pinto-da-Rocha

**Affiliations:** 1 Department of Zoology, Universidade de São Paulo (USP), São Paulo (SP), Brazil Universidade de São Paulo (USP) São Paulo Brazil; 2 Department of Biology and Zoology, Universidade Federal de Mato Grosso (UFMT), Cuiabá (MT), Brazil Universidade Federal de Mato Grosso (UFMT) Cuiabá Brazil; 3 Universidade Federal do Piauí, Campus Almícar Ferreira Sobral, Floriano (PI), Brazil Universidade Federal do Piauí Floriano Brazil

**Keywords:** Chromosomal rearrangements, chromosome, fluorochrome, heterochromatin, meiosis, silver impregnation

## Abstract

*
Bothriurus* Peters, 1861 is one of the most diverse genera within the family Bothriuridae. However, to date, only five species have been analyzed using a cytogenetic approach. In this study, for the first time, two populations of *Bothriurus asper* Pocock, 1893 and nine populations of *Bothriurus rochai* Mello-Leitão, 1932, two species from northeastern Brazil, were analyzed with respect to diploid number, chromosomal behavior during meiosis, and the localization of heterochromatin and nucleolus organizer regions (NORs). For *B. asper*, a diploid number of 2n = 30 was recorded in geographically distant populations, whereas *B. rochai* exhibited intraspecific variation in diploid number (2n = 16 and 2n = 18), representing the lowest diploid numbers ever reported for the family Bothriuridae. Despite the variability in diploid number, the number and localization of NORs remained stable among the populations of *B. rochai*. When comparing heterochromatin patterns between the two species, larger blocks of constitutive heterochromatin were observed in *B. asper* than in *B. rochai*. Variation in the amount of heterochromatin among populations of *B. rochai* was also observed; in this case, the population with the lowest amount of heterochromatin also exhibited the greatest variation in post-pachytene cell configurations. This is the first study to cytogenetically analyze multiple populations of species within the genus *Bothriurus*, and it significantly expands the karyotypic information available for scorpions with monocentric chromosomes.

## ﻿Introduction

Scorpions represent the third most extensively well-studied order of arachnids from a cytogenetic perspective (Šťáhlavský et al. 2018). They have a synaptic and achiasmatic male meiosis, whose correct segregation of chromosomes is guaranteed by the permanence of the synaptonemal complex until later phases of meiosis I (Schneider et al. 2009a, 2009b; Almeida et al. 2019) and different types of chromosomes. With the exception of Buthidae, the only scorpion family that presents holocentric chromosomes, the other ten scorpion families that have been cytogenetically characterized (Bothriuridae, Chactidae, Chaerilidae, Euscorpiidae, Iuridae, Liochelidae, Scorpionidae, Scorpiopidae, Urodacidae, Vaejovidae) possess monocentric chromosomes (Melters et al. 2012; Schneider et al. 2009a, 2024). However, most of these families have at most 10 species that have been studied (Almeida et al. 2023; Schneider et al. 2024), and together, these families account for less than 40% of the cytogenetic data available for scorpions.

Chromosomal rearrangements are the main drivers of karyotypic differences (Mezzasalma et al. 2024) and can generate polymorphism in natural populations (White 1973; Joron et al. 2011). Fissions/fusions rearrangements and translocations have been reported in different scorpion species (e.g. Schneider et al. 2009a, 2009b; Mattos et al. 2013; Adilardi et al. 2020; Lima et al. 2023), and are the primary factors responsible for the high intraspecific karyotype variability and the diverse chromosomal configurations observed during meiosis (Mattos et al. 2018). The identification and characterization of polymorphism expand our understanding of chromosomal evolution (e.g. Jacobina et al. 2009; Mattos et al. 2013; Adilardi et al. 2014, 2016, 2020; Almeida et al. 2017; Ubinski et al. 2018; Lima et al. 2023).

In general, cytogenetics is a powerful tool for the characterization of natural groups and has proven effective in characterizing different scorpion lineages (Ojanguren-Affilastro et al. 2017; Šťáhlavský et al. 2018; Štundlová et al. 2019) as well as in identifying population-level polymorphism (e.g. Mattos et al. 2013, 2018; Ubinski et al. 2018; Adilardi et al. 2020; Lima et al. 2020, 2023). Thus, different cytogenetic markers have been widely applied in scorpion studies for the characterization of species and populations. Among the most frequently used are constitutive heterochromatin regions and nucleolus organizer regions (NORs).

The pattern of distribution and amount of constitutive heterochromatin is variable within Scorpiones and can assist in distinguishing species and populations (e.g. Mattos et al. 2013; Adilardi et al. 2015; Almeida et al. 2017; Ojanguren-Affilastro et al. 2017; Lima et al. 2023). Although the number and position of NORs appear to be stable for some Buthidae species (Šťáhlavský et al. 2020), as observed in representatives of the genera *Ischnotelson* Esposito, Yamaguti, Souza, Pinto da Rocha et Prendini, 2017, *Jaguajir* Esposito, Yamaguti, Souza, Pinto da Rocha et Prendini, 2017, *Physoctonus* Mello-Leitão, 1934, *Rhopalurus* Thorell, 1876, and *Tityus* C. L. Koch, 1836 (Adilardi et al. 2013, 2014; Mattos et al. 2013, 2014; Ubinski et al. 2018), other genera have shown variation in the number or position of these regions, for example, species of *Compsobuthus* Vachon, 1949, *Hottentotta* Birula, 1908, and *Reddyanus* Vachon, 1912 (Šťáhlavský et al. 2020). These data highlight the effectiveness of this marker in distinguishing certain scorpion taxa.

The family Bothriuridae comprises 168 described species (Rein, 2025). However, only three genera (*Brachistosternus* Pocock, 1893, *Bothriurus* Peters, 1861, and *Timogenes* Simon, 1880) and less than 6% of the species of this family have had their chromosomes studied (Schneider et al. 2024). In Brazil, the genus *Bothriurus* is the most diverse, currently comprising 17 described species (Rein 2025). However, only five species of this genus have been cytogenetically characterized, and only two of them, *B. araguayae* Vellard, 1934 (2n = 42 and 44) (Ferreira 1968; Schneider et al. 2009b) and *Bothriurus* sp. (2n = 36) (Piza 1947) belong to the Brazilian fauna.

Additionally, only *B. araguayae* and *B. rochensis* San Martin, 1965 (2n = 46) have been characterized in terms of chromosomal behavior during meiosis. These species exhibited distinct diploid numbers (including intraspecific variation observed among populations of *B. araguayae*), as well as differences in the number of NORs (4 and 6, respectively), the localization of heterochromatin, and chromosome morphology (Schneider et al. 2009b). These results reinforce the potential of cytogenetic markers in the characterization of *Bothriurus* species and, consequently, in biodiversity assessment. Chromosomal differences have also highlighted the utility of cytogenetic data for species-level taxonomy, as observed in representatives of Scorpiopidae (Šťáhlavský et al. 2021).

*Bothriurus asper* Pocock, 1893 and *B. rochai* Mello-Leitão, 1932 are species widely distributed in northeastern Brazil (Maury 1982; Lourenço 2000; Mattoni et al. 2003; Santos-da-Silva et al. 2017). Both belong to distinct species groups named after them, which still face significant taxonomic challenges (Santos-da-Silva et al. 2017). These species groups are poorly understood from taxonomic, karyological, phylogenetic, and ecological perspectives.

In this context, cytogenetic analyses not only contribute to understanding the impact of chromosomal rearrangements on karyotype evolution but also enable the identification of potential diagnostic chromosomal characters and the detection of population-level polymorphism. Understanding the chromosomal organization of the genome is essential for identifying processes related to population divergence, adaptation, and, in some cases, even speciation events (Sheffer et al. 2021). Evolutionary processes may be accompanied by changes in genome organization and not solely by point mutations in the DNA sequence (Štundlová et al. 2019).

The absence of information on inter- and intraspecific karyotypic variability in these two *Bothriurus* species makes them suitable models for cytogenetic characterization. Furthermore, this study addresses a broader gap in cytogenetic data available for species of the genus *Bothriurus*. Thus, the present work characterized different populations of *B. asper* and *B. rochai* with respect to diploid number and chromosomal behavior during meiosis, with the aim of expanding cytogenetic knowledge for the group, as well as enabling the future integration of the data obtained here into broader biodiversity research.

## ﻿Material and methods

### ﻿Sampling

The number of specimens analyzed in this study and their respective collection sites in northeastern Brazil are listed in Table 1 (see Suppl. material 1: table S1, fig. S1). All specimens were deposited in the Natural History Collection of the Federal University of Piauí, Floriano, Brazil (CHNUFPI; curator: J.F. Vilela).

**Table 1.** *
Bothriurus* species analyzed in this study, including the number of individuals and their collection localities. CE- Ceará, PB- Paraíba, PE- Pernambuco, PI-Piauí, RN- Rio Grande do Norte.

Species	Number of individuals	Collection localities
*** Bothriurus asper***		
	4♂	Teresina-PI (4°54'13.2"S, 42°47'27"W)
	1♂	Igarassu-PE (7°46'33"S, 34°56'54"W)
*** Bothriurus rochai***		
	2♂	Apodi -RN (5°35' 27"S, 37°49' 39"W)
	3♂	Cajazeiras-PB (6°52' 37"S, 38°37'03"W)
	2♂	Maturéia-PB (7°16'18"S, 37°18'5.1"W)
	1♂	Icó-CE (6°24'46"S, 39°03'55"W)
	3 ♂	Quixadá-CE (4°55'57"S, 39°10'22"W)
	8♂	Brasileira-PI (4°06'28"S, 41°42'04"W)
	2♂	João Câmara-RN (5°34'02"S, 35°55'03"W)
	2♀	Floriano-PI (6°46 21"S, 43°03'48"W)
	9♂	São Raimundo Nonato-PI (8°52'45"S, 42°43'08"W)

### ﻿Cytogenetic preparations

Slides were prepared from the gonads of adult individuals according to the technique described by Schneider et al. (2009a). The slides were stained with 3% Giemsa solution for 12 minutes to perform the initial characterization of diploid number, chromosomal configurations in meiotic cells and chromosome morphology. The nomenclature for chromosome morphology followed Levan et al. (1964). However, only two categories were considered: meta/submetacentric for chromosomes with two arms and subtelo/acrocentric for chromosomes with only one clearly visible chromosome arm. This is because the analyses were performed in metaphase II cells, in which the chromosomes are more condensed.

The diploid set length (**DSL**) was evaluated for *B. rochai* populations. At least three post-pachytene cells per population, exhibiting similar levels of chromatin condensation, were analyzed using ImageJ (Image Processing and Analysis in Java) (see Suppl. material 1: fig. S2), developed by the Research Services Branch of the U.S. National Institute of Mental Health. All cells were measured in micrometers, including all bivalents as well as the chromosomes constituting the chromosomal chain, when present.

A generalized linear model (**GLM**) with Gaussian distribution of errors was fitted to compare the DSL among cells with different chromosomal arrangements. Model adequacy was evaluated by manually examining the dispersion parameters. A contrast analysis was conducted using the ‘coms’ function from the R package ‘RT4Bio’ (Reis-Júnior et al. 2015), to identify which chromosomal arrangement groups differed significantly. A figure was generated using the ‘ggplot2’ package (Wickham 2016).

To detect active nucleolus organizer regions (**NORs**), silver ion impregnation was performed according to the method described by Howell and Black (1980) and constitutive heterochromatin regions were identified following the protocol described by Sumner (1972), with a modified incubation time of 30 seconds in 5% barium hydroxide octahydrate solution at 60 °C.

Additionally, to visualize AT- and GC-rich chromatin regions, some slides were stained with 4’-6-diamidino-2-phenylindole (**DAPI**), chromomycin A3 (**CMA**₃), and distamycin A, following Schweizer (1976). All chromosomal preparations were photographed using a Zeiss Axio Imager A2 light microscope with Zen 3.4 software and an Olympus BX51 fluorescence microscope with DP Controller software, using appropriate filters for fluorescent dyes.

## ﻿Results

**Figure 1.**
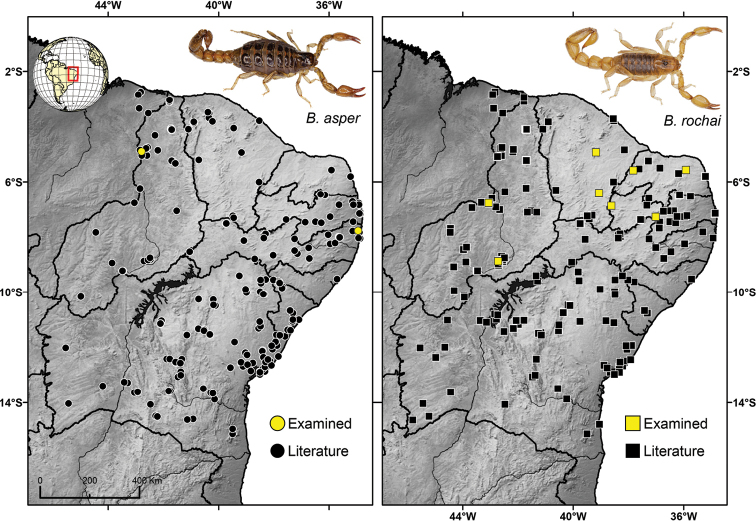
Distribution and collection localities in Brazil of *Bothriurus asper* and *Bothriurus rochai* analyzed in this work.

*Bothriurus asper* included two analyzed populations (Fig. 1, Table 1), with the cytogenetic description comprising specimens from the type locality, Igarassu-PE. In contrast, a total of nine populations of *B. rochai* were characterized, one of them from its type locality (vaguely known only as the state of Ceará; Maury, 1982) (Fig. 1, Table 1). Cytogenetic analyses revealed that both *B. asper* and *B. rochai* possess monocentric chromosomes, with clearly defined primary constrictions and centromeric regions.

### ﻿Chromosomal characterization

**Figure 2.**
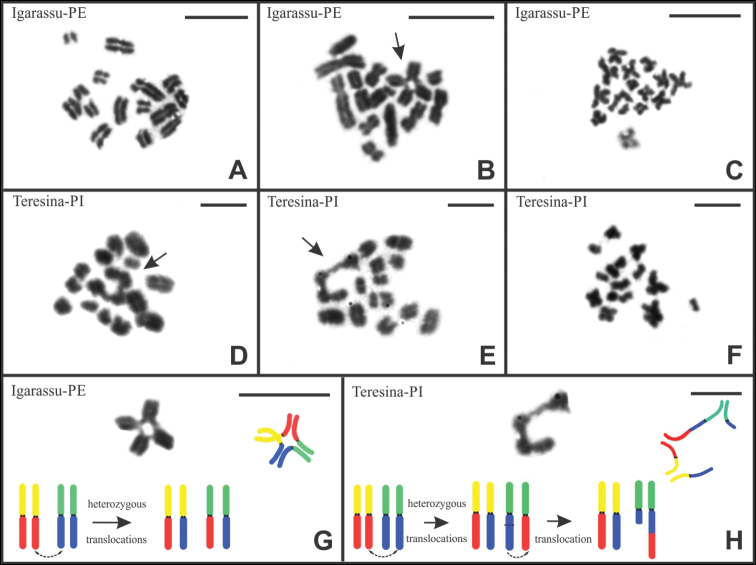
Testicular cells of *B. asper* stained with Giemsa. Post-pachytene cell with 15 bivalents (15II) (**A**). Post-pachytene cells with 13 bivalents and a chain of four chromosomes (13II + CIV) (**B, D, E**). Meiotic metaphases with 15 chromosomes (**C, F**). Diagrams illustrating the chromosomal rearrangements (**G, H**). Arrows = chromosomal chains. Scale bars: 10 μm.

In the analyzed specimens of *B. asper*, no mitotic metaphase cells were observed. Therefore, the diploid number 2n = 30 was determined based on the analysis of post-pachytene and metaphase II cells. In the specimen from Igarassu-PE, post-pachytene nuclei exhibited two distinct configurations: 15 side-by-side bivalents (29.5% of the cells), with no evidence of chiasmata (Fig. 2A), and 13 bivalents plus one chromosomal chain composed of four elements (70.5% of the cells), arranged in a cross-shaped configuration (Fig. 2B). Metaphase II cells revealed the presence of 15 chromosomes (n = 15), including 11 meta/submetacentric and four subtelo/acrocentric chromosomes (Fig. 2C).

In the four specimens from Teresina-PI, only post-pachytene cells displaying a chromosomal chain composed of four elements (13II + CIV) were observed. However, unlike the individual from Igarassu-PE, all cells showed an open quadrivalent association (Fig. 2D, E). Finally, metaphase II cells revealed a haploid number of n = 15, consisting of five meta/submetacentric and ten subtelo/acrocentric chromosomes (Fig. 2F). Schematic representations of multivalent associations, for both species, can be seen in the Fig. 2G, H.

**Figure 3.**
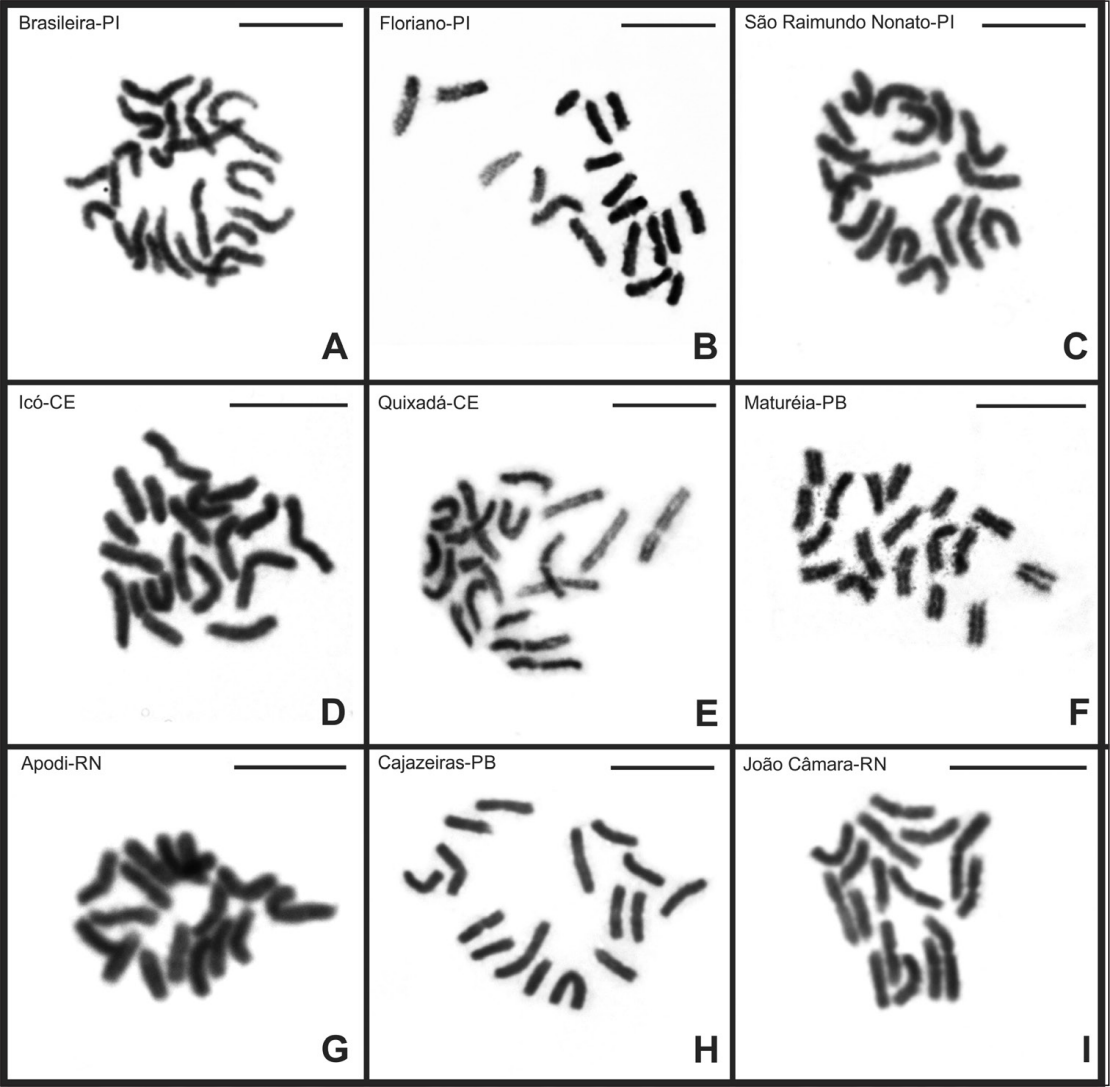
Mitotic metaphase cells of *B. rochai* stained with Giemsa. Mitotic metaphase cells showing 2n = 18 (**A, B, C, D, E, F**). Mitotic metaphase cells showing 2n = 16 (**G, H, I**). Scale bars: 10 µm.

**Figure 4.**
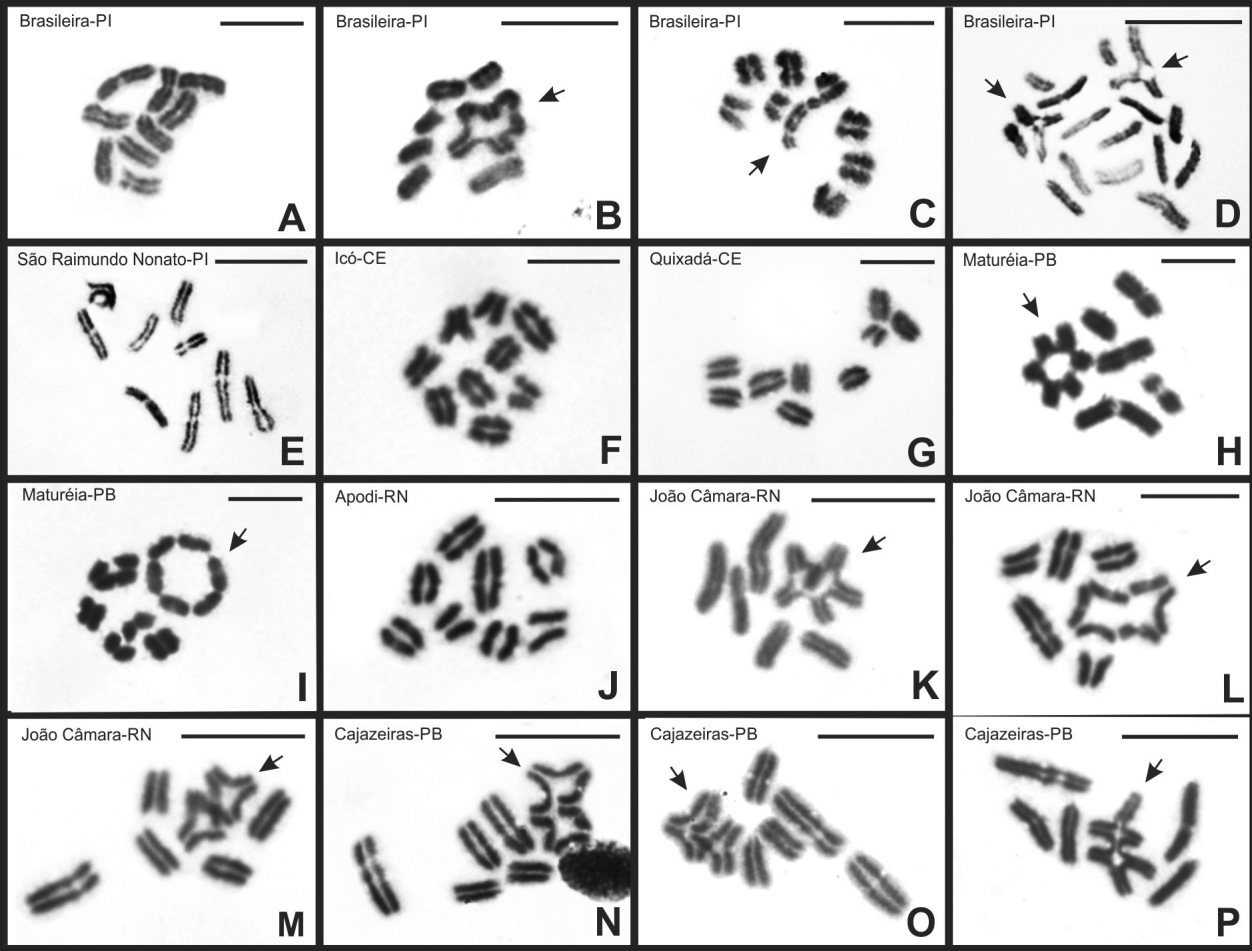
Post-pachytene cells of *B. rochai* stained with Giemsa. Nine bivalents (**A, E, F, G**). Eight bivalents (**J**). 6II + CVI (**B, H, I**). 7II + CIV (**C**). 7II + CIV (**D**) (two cells shown). 5II + CVI (**K, L, M, N, O, P**). Arrow = chromosomal chain. Scale bars: 10 μm.

**Figure 5.**
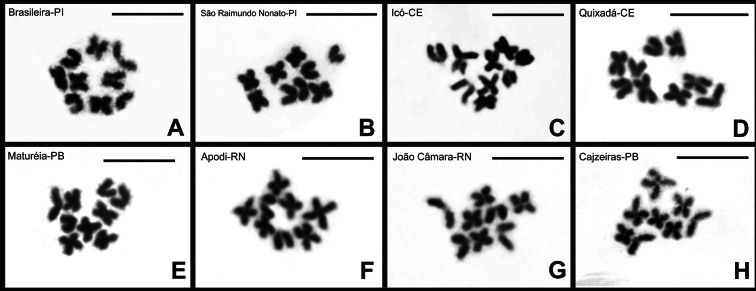
Metaphase II cells of *B. rochai* stained with Giemsa. Cells with n = 9 (**A, B, C, D, E**). Cells with n = 8 (**F, G, H**). Scale bars: 10 μm.

The cytogenetic characterization of *B. rochai* revealed intraspecific variation in diploid number (Figs 3–5) (Suppl. material 1: fig. S1). Mitotic metaphase cells from males allowed the determination of a diploid number of 2n = 18 for individuals from Brasileira-PI, Floriano-PI, São Raimundo Nonato-PI, Icó-CE, Quixadá-CE, and Maturéia-PB (Fig. 3A–F), and 2n = 16 for the populations from Apodi-RN, Cajazeiras-PB, and João Câmara-RN (Fig. 3G–I). Floriano-PI was the only population from which only female specimens were obtained; these individuals showed 18 chromosomes in mitotic metaphase cells, consistent with most of the analyzed populations (Fig. 3B).

Of the eight specimens examined from Brasileira-PI, three exhibited post-pachytene nuclei composed exclusively of nine bivalents (Fig. 4A), while the remaining five showed variation in chromosomal configurations. These latter specimens presented cells with 9II, cells with six bivalents and one chromosomal chain composed of six chromosomes (6II + CVI) (Fig. 4B), and a few cells with seven bivalents and one quadrivalent chromosomal chain (7II + CIV) (Fig. 4C, D). For all specimens from São Raimundo Nonato-PI, Icó-CE, and Quixadá-CE, only cells with nine side-by-side bivalents were observed, with no evidence of chiasma (Fig. 4E–G).

In the specimens from Maturéia-PB, post-pachytene cells also revealed the presence of six bivalents and a chromosomal chain composed of six chromosomes (6II + CVI) (Fig. 4H, I) in both individuals analyzed. However, some degree of instability was observed in the bivalent configurations within this population. While in some cells, five isolated bivalents were observed, with the ends of two pairs positioned very close to each other (Fig. 4H), other cells appeared to show a terminal association between the two bivalents (Fig. 4I).

Among the populations with a diploid number of 2n = 16, only in specimens from Apodi-RN did all post-pachytene nuclei exhibit exclusively bivalents (8II) (Fig. 4J). In contrast, all cells from the João Câmara-RN and Cajazeiras-PB populations displayed five large bivalents and a chromosomal chain composed of six medium-sized chromosomes (5II + CVI) (Fig. 4K–P). However, varying degrees of association between the chromosomes forming the chain were observed. While in some cells only the terminal and subterminal regions of the chromosomes were associated (Fig. 4L), in others, an extensive portion of the chromosomes was synapsed (Fig. 4P).

Metaphase II cells revealed haploid numbers of n = 9, with five meta/submetacentric and four subtelo/acrocentric chromosomes in specimens from Brasileira-PI, São Raimundo Nonato-PI, Icó-CE, Quixadá-CE, and Maturéia-PB (Fig. 5A–E), n = 8 with six metacentric and two acrocentric chromosomes in specimens from Apodi-RN and Cajazeiras-PB (Fig. 5F, H), and four meta/submetacentric and four acrocentric chromosomes in specimens from João Câmara-RN (Fig. 5G).

To assess differences in chromosome size, DSL was measured in post-pachytene cells with comparable levels of chromatin condensation. The average DSL was 2.50 µm in individuals with 2n = 18 and 1.59 µm in those with 2n = 16, with specific values for cells containing only bivalents and those forming chromosomal chains, summarized in Table 2.

**Figure 6.**
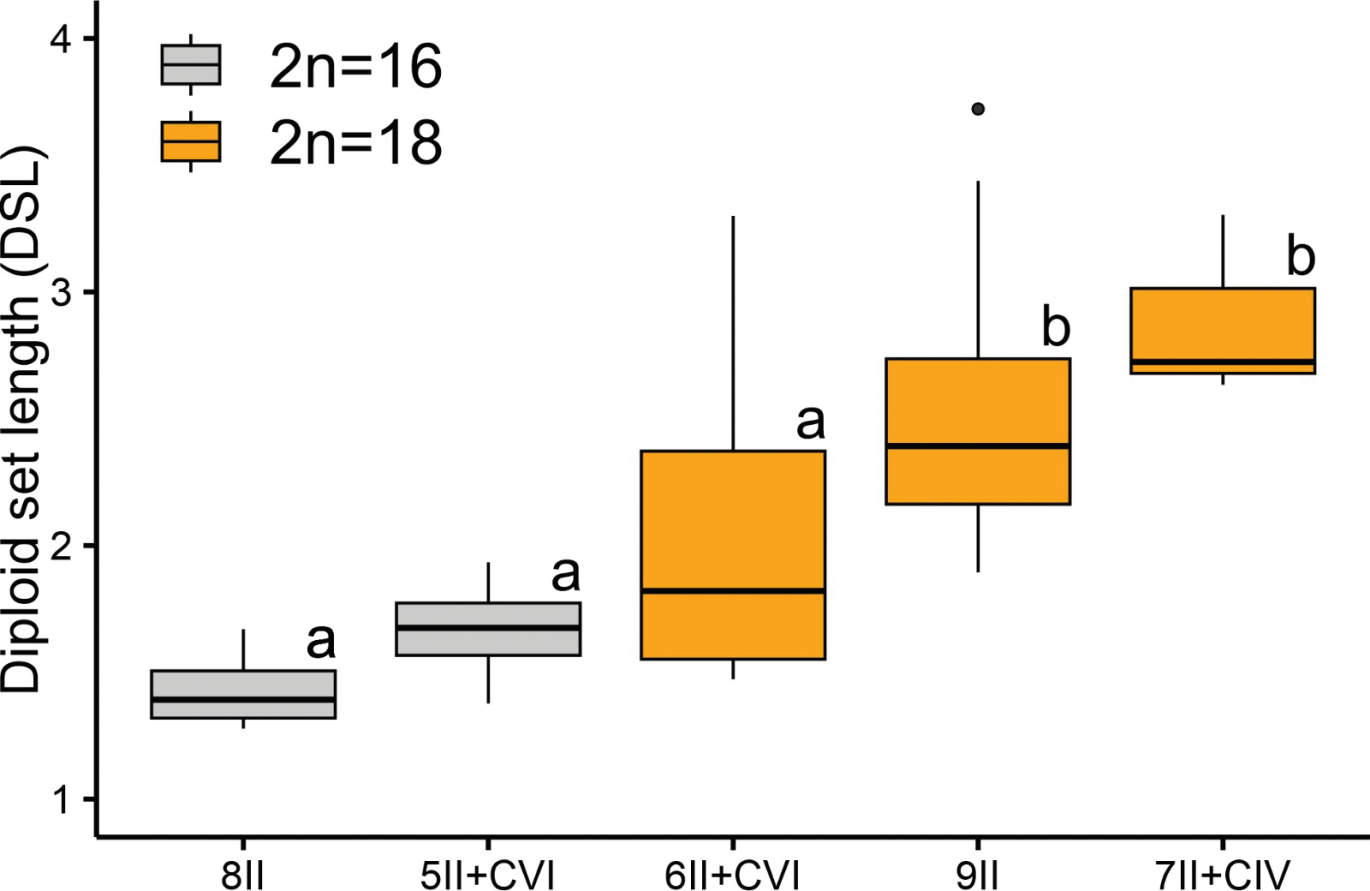
Comparisons of diploid set length among 2n = 16 (gray boxes) and 2n = 18 (orange boxes) specimens of *Bothriurus rochai*. Different letters above the boxes denote statistically distinct groups.

The analysis revealed significant variation in DSL among chromosome organizations (residual deviance = 8.101, d.f. = 31; F = 9.259, p < 0.001). Post-pachytene cells of specimens with 2n = 16 and those with 2n = 18 organized as 6II + CVI exhibited smaller chromosomes, ranging from small to medium sizes. In contrast, specimens with 2n = 18 organized as 9II or 7II + CIV showed larger DSL values. Additionally, the size of the bivalents and the chromosomes involved in multivalent associations were slightly smaller in the 2n = 16 and 6II + CVI groups than in the 9II and 7II + CIV groups (Figs 4, 6).

**Table 2.** Results of the cytogenetic characterization of *B. asper* and *B. rochai* species and the number of cells analyzed. II = bivalents. Number of cells in parentheses. DSL = diploid set length (average value), C = chain. *DSL values from these cells were combined with populations that exhibited the same configurations.

Species	Populations	Diploid number (2n)	chromosome configurations	% and number of cells	Haploid number (n)	DSL (µm)
*** Bothriurus asper***						
	Igarassu-PE	2n = 30	15II	29.5 (5)	n = 15 (8)	
			13II+CIV	70.5 (12)		
	Teresina-PI	2n = 30	13II+CIV	100 (30)	n = 15 (5)	
*** Bothriurus rochai***						
	Brasileira-PI	2n = 18 (17)	9II	64 (50)	n = 9 (21)	2.52
			7II+CIV	23 (18)		2.88
			6II+CVI	13 (10)		2.10
	São Raimundo Nonato-PI	2n = 18 (26)	9II	100 (95)	n = 9 (74)	*
	Floriano-PI	2n = 18 (3)	_	_	_	
	Apodi -RN	2n = 16 (3)	8II	100 (20)	n = 8 (9)	1.45
	João Câmara-RN	2n = 16 (60)	5II+CVI	100 (26)	n = 8 (7)	1.67
	Quixadá-CE	2n = 18 (12)	9II	100 (7)	n = 9 (6)	*
	Icó-CE	2n = 18 (5)	9II	100 (29)	n = 9 (5)	*
	Cajazeiras-PB	2n = 16 (38)	5II+CVI	100 (18)	n = 8 (12)	*
	Maturéia-PB	2n = 18 (4)	6II+CVI	100 (25)	n = 9 (11)	*

### ﻿Localization of nucleolus organizer regions (NORs)

**Figure 7.**
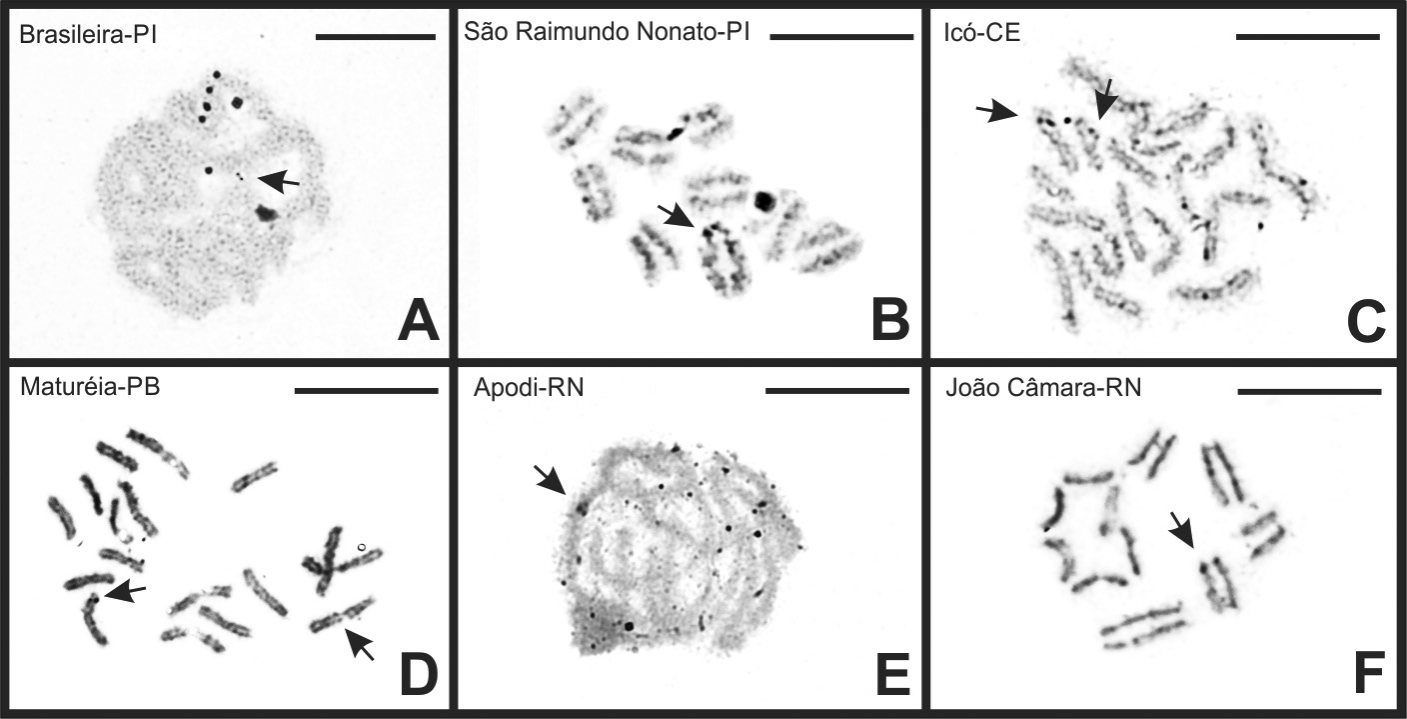
*
B. rochai* cells subjected to silver ion impregnation. Note the nuclear organizing regions (arrows) present in the terminal region of two chromosomes (**A, B, C, D, E, F**). Scale bars: 10 µm.

For *B. rochai*, chromosome preparations subjected to silver ion impregnation indicated the presence of active nucleolus organizer regions at the terminal region of one chromosome pair (Fig. 7). For specimens with diploid number 2n = 18, small markings were observed in pachytene cells from Brasileira-PI (Fig. 7A), at the terminal regions of two chromosomes in specimens from São Raimundo Nonato-PI (Fig. 7B) and Maturéia-PB (Fig. 7D). For Icó-CE, two markings were also observed, both in the subterminal region of the chromosomes (Fig. 7C).

For individuals with diploid number 2n = 16, small markings were observed in pachytene cells from Apodi-RN (Fig. 7E) and at the terminal regions of two chromosomes in specimens from João Câmara-RN (Fig. 7F). For the other populations of *B. rochai*, as well as for the two populations of *B. asper*, no positive markings were observed.

### ﻿Localization of constitutive heterochromatin and CMA_3_/DA/DAPI staining

**Figure 8.**
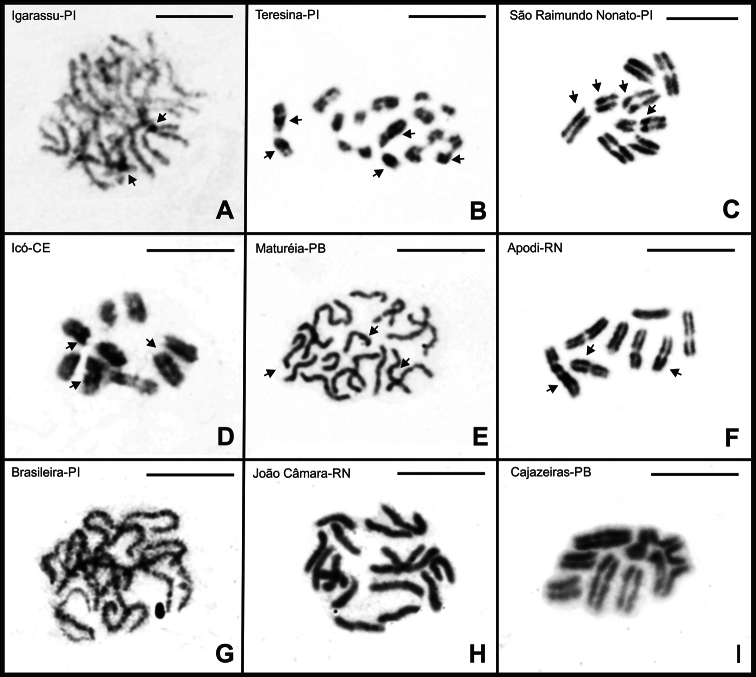
Testicular cells of *B. asper* and *B. rochai* subjected to the C-banding technique. Cells of *B. asper* (**A, B**) and *B. rochai* (**C–I**). Arrows indicate regions of constitutive heterochromatin. Scale bars: 10 µm.

Slides from specimens of both *B. asper* populations were subjected to C-banding and CMA₃/DA/DAPI fluorochrome staining. However, only the C-banding technique yielded positive results. This species exhibited constitutive heterochromatin blocks detectable in pachytene cells, as well as in the terminal and pericentromeric regions of certain chromosomes in post-pachytene cells—specifically in four bivalents and in chromosomes involved in the multivalent chain (Fig. 8A, B). Since this technique could not be applied to metaphase cells, it was not possible to precisely identify which chromosomes carried these heterochromatic regions.

For *B. rochai*, C-banding was applied to all populations except for individuals from Floriano-PI and Quixadá-CE, which exhibited few dividing cells or did not yield positive results after the technique was applied. The remaining populations displayed variation in the distribution patterns of heterochromatin. Specimens from São Raimundo Nonato-PI and Icó-CE exhibited small blocks of constitutive heterochromatin in the terminal regions of three bivalents (Fig. 8C, D). In Maturéia-PB specimens, small terminal blocks were also observed, along with faint pericentromeric markings (Fig. 8E). Notably, in Apodi-RN, the only 2n = 16 population with positive heterochromatin staining, larger blocks were detected in the terminal and pericentromeric regions of three bivalents (Fig. 8F). No positive C-banding signals were detected in specimens from Brasileira-PI, João Câmara-RN, and Cajazeiras-PB (Fig. 8G, H, I).

**Figure 9.**
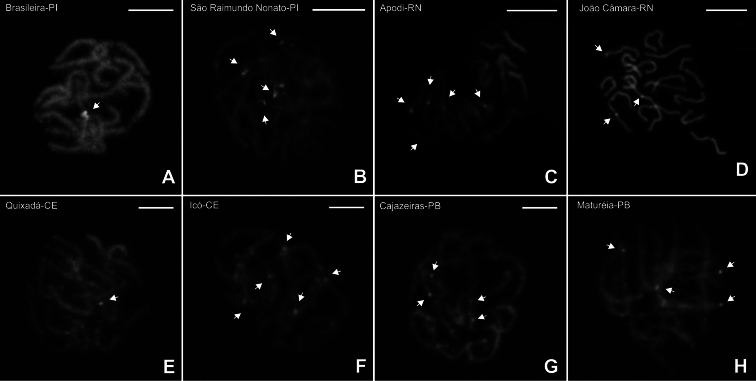
*
B. rochai* cells subjected to triple staining CMA3/DA/DAPI, highlighting the DAPI (AT-) rich regions (arrows). Scale bars: 10 µm.

For *B. rochai*, CMA₃/DA/DAPI staining revealed a greater number DAPI bright signals compared to C-banding. However, the chromosomes of all individuals were homogeneously CMA_3_ stained, with no specific brilliant region. A prominent DAPI-positive (AT-rich) block was detected in pachytene cells from specimens of Brasileira-PI (Fig. 9A), while small AT-rich regions were observed in the terminal and interstitial segments of bivalents in specimens from São Raimundo Nonato-PI (Fig. 9B), Apodi-RN (Fig. 9C), João Câmara-RN (Fig. 9D), Quixadá-CE (Fig. 9E), Icó-CE (Fig. 9F), Cajazeiras-PB (Fig. 9G), and Maturéia-PB (Fig. 9H).

## ﻿Discussion

For the first time, the species *B. asper* and *B. rochai* were described with respect to diploid number, chromosomal behavior during meiosis, and the localization of heterochromatin and nucleolar organizer regions. Both species exhibited relatively low diploid numbers, with 2n = 16 and 2n = 18 observed in populations of *B. rochai*, the lowest numbers ever reported for the family (Schneider et al. 2024). Higher chromosome numbers, such as 2n = 42 and 44 for *B. araguayae* (Ferreira 1968; Schneider et al. 2009b), 2n = 48 for *B. flavidus* Kraepelin, 1911, 2n = 50 for *B. prospicuus* Mello-Leitão, 1934 (Schneider et al. 2024), and 2n = 46 for *B. rochensis* (Schneider et al. 2009b), have previously been described. This characteristic also appears to be common in other taxa of the family Bothriuridae, such as *Timogenes elegans* Mello-Leitão, 1931 (2n = 48), and four species of *Brachistosternus* Pocock, 1893, which showed 2n = 42 and 2n = 46, with the exception of *Brachistosternus alienus* Lonnberg, 1898, which exhibited intraspecific variation (2n = 46 and 2n = 28) (Schneider et al. 2024).

In this work, for *B. asper*, a diploid number of 2n = 30 was described for geographically distant populations located in different biomes (Atlantic Forest and Caatinga). The fact that the same number was found in distant populations is strong evidence that this is the diploid number for the species. Intraspecific variation has been reported for at least 23 distinct scorpion species (Schneider et al. 2024). However, constant chromosome numbers have also been described for different genera and species of the family Buthidae (e.g. Sadílek et al. 2015; Ubinski et al. 2018; Kovařík et al. 2020).

Considering the absence of intraspecific variability in the diploid number, the formation of multivalent associations in *B. asper* may be attributed to the occurrence of reciprocal chromosomal translocations. Heterozygous reciprocal translocations and fission/fusion rearrangements are consistently invoked as being responsible for the origin of multivalent chromosomal associations during meiosis I in scorpions (e.g. Schneider et al. 2009a; Mattos et al. 2013, 2018; Adilardi et al. 2016, 2020). Translocations between non-homologous elements may have originated from the closed quadrivalent chromosomal chain observed in the individual from Igarassu-PE. Multivalent associations resulting from reciprocal translocations have already been described for other scorpion species with monocentric chromosomes (Almeida et al. 2023; Lima et al. 2023), including *B. araguayae*, which showed a heterozygous translocation involving two arms of non-homologous chromosomes (Schneider et al. 2009b).

For the specimens from Teresina-PI, the presence of an open configuration also seems to result from two translocations. In this case, a reciprocal translocation between non-homologous chromosomes followed by a non-reciprocal translocation would have transferred a fragment from the end of one chromosome to the interstitial region of another element. This is the simplest hypothesis considering the number of chromosomes involved in the open chain.

The persistence of unsynapsed regions, visualized in post-pachytene cells as open configurations, is uncommon; generally, multivalent chromosomal associations appear closed, with synapsed regions forming a ring (Mattos et al. 2018). Nevertheless, this type of open formation or the presence of gaps has already been observed in buthid genera such as *Tityus* (Mattos et al. 2018), *Rhopalurus*, *Ischnotelson* (Ubinski et al. 2018), and *Heterometrus* Pocock, 1893 (Rajasekarasetty, 1979). In these taxa, however, such configurations with unsynapsed fragments seem to be related to later stages of prophase I (Mattos et al. 2018; Ubinski et al. 2018).

Unlike *B. asper*, *B. rochai* exhibited variation in diploid number. Interpopulational chromosomal number variations have been attributed to fission/fusion-type rearrangements in different scorpion species with holocentric chromosomes (e.g. Schneider et al. 2009a; Mattos et al. 2013, 2018; Almeida et al. 2017; Ojanguren-Affilastro et al. 2017; Adilardi et al. 2016, 2020; Šťáhlavský et al. 2020) and monocentric chromosomes (e.g. Štundlová et al. 2019; Almeida et al. 2023). However, it is difficult to determine whether the 2n = 16 karyotype originated from 2n = 18 by chromosomal fissions, or if 2n = 18 originated from 2n = 16 by chromosomal fusions. The smaller DSL observed in 2n = 16 and in 2n = 18 configurations formed by 6II + CVI suggests that the variation in these chromosome arrangements may involve a more complex scenario than simple fusions or fissions. In addition to affecting chromosome number, rearrangements are a major force in altering chromosome size (Schubert 2007; Li et al. 2011). When comparing the size of the chromosomal set in individuals with different diploid numbers, significant differences were observed, suggesting loss or gain of genetic material, resulting from chromosomal deletions or duplications. To determine which is the ancestral state, phylogeographic or phylogenetic analyses would allow a more robust reconstruction of the ancestral number.

Nevertheless, the presence of chromosomal associations in post-pachytene cells indicates that these karyotypes did not arise solely through these types of rearrangements. Chromosomal configurations formed by four and six chromosomes are results of reciprocal translocations, indicating that independent rearrangements are responsible for the chromosomal characteristics of this species. Chromosomal rearrangements can change chromosomal architecture and drive genetic differentiation (Ren et al. 2018; Xia et al. 2020). The structural differences observed in the karyotypes of both species indicate a possible dynamic evolutionary change in the genome organization of populations (Just et al. 2022).

Nucleolus organizer regions include the main clusters of ribosomal RNA genes (Potapova and Gerton 2019). This marker is frequently used in studies of chromosomal evolution in scorpions (e.g. Schneider et al. 2009a, 2009b; Schneider and Cella 2010; Mattos et al. 2013, 2014, 2018; Adilardi et al. 2014, 2015, 2016; Šťáhlavský et al. 2018, 2021; Ubinski et al. 2018; Lima et al. 2020). However, in species with monocentric chromosomes, it has been applied to only 31 species (Schneider et al. 2009b; Šťáhlavský et al. 2018, 2021; Štundlová et al. 2019; Almeida et al. 2023; Lima et al. 2023), out of the 95 that have been cytogenetically described regarding this feature.

Despite variability in diploid number, the amount and localization of NORs remained stable among populations of *B. rochai*. Having a single pair of NORs matches the most frequent pattern reported for scorpions, including species with monocentric chromosomes already analyzed (Schneider et al. 2009b; Šťáhlavský et al. 2018, 2021; Štundlová et al. 2019; Almeida et al. 2023; Lima et al. 2023). A single pair of NORs at the terminal region of chromosomes appears to be the ancestral condition for arachnids (Forman et al. 2013) and has remained conserved during chromosomal evolution of these populations. The non-random distribution of rDNA sites may be related to structural and organizational aspects, such as the amount of nuclear DNA and the dynamics of chromosomes during interphase (Roa et al. 2012).

When comparing heterochromatin patterns between the two species, larger blocks of constitutive heterochromatin are apparent in *B. asper* than in *B. rochai*. However, both species also exhibited heterochromatic blocks at the terminal regions of the chromosomes. The amount and localization of heterochromatin is variable among scorpion species of the family Buthidae (Adilardi et al. 2013; Mattos et al. 2013), but for scorpions with monocentric chromosomes, a small amount of heterochromatin restricted to the pericentromeric region is the most frequent pattern (Adilardi et al. 2013; Plíšková et al. 2016; Almeida et al. 2023). Even so, the localization pattern observed in both *Bothriurus* species is similar to that described for another species of the same genus, *B. rochensis*, which also showed markings at the terminal regions as well as pericentromeric markings (Schneider et al. 2009b).

In scorpions, the amount of heterochromatin appears to be inversely proportional to the frequency of chromosomal rearrangements, that is, indices of chromosomal rearrangements are higher in species with a lower amount of heterochromatin (Mattos et al. 2013). For *B. rochai*, the population that showed the smallest amount of heterochromatin (Brasileira-PI) also exhibited the greatest variation in configurations in post-pachytene cells. This comparison can also be made between species; *B. asper*, which exhibited larger heterochromatic blocks, showed only a single distinct configuration in post-pachytene cells. Finally, the absence of C-banding technique markings in specimens from the populations of Brasileira-PI, João Câmara-RN, and Cajazeiras-PB may be related to the fact that these populations have few or very small heterochromatin blocks, which likely makes them difficult to observe using this technique, as described for the species *Neochactas* sp. and *Chactopsis amazonica* Lourenço et Francke, 1986 (Lima et al. 2023).

The results obtained in this study broaden our understanding of karyotypic diversity in the genus *Bothriurus*, as well as the cytogenetic information available for scorpions with monocentric chromosomes. The stability of the diploid number in *B. asper*, in contrast to the variation observed in *B. rochai*, suggests distinct trajectories of karyotype evolution within the group, in which chromosomal rearrangements, especially translocations and fission/fusion events, play a central role. Additionally, the differences in the amount and distribution of heterochromatin, associated with the conservation of a single pair bearing the nucleolar organizer regions (NORs), indicate that, despite the structural differences observed, some elements of genomic organization remain conserved.

## Supplementary materials

Supplementary material 1 Supporting information Data type docx Explanation note List of specimens of *Bothriurus asper* and *Bothriurus rochai* used in this study including identification code and colle ction location of specimen. https://binary.pensoft.net/file/1465064This dataset is made available under the Open Database License (http://opendatacommons.org/licenses/odbl/1.0/). The Open Database License (ODbL) is a license agreement intended to allow users to freely share, modify, and use this Dataset while maintaining this same freedom for others, provided that the original source and author(s) are credited.Juliana F. de Lima, Marielle C. Schneider, Leonardo S. Carvalho, Ricardo Pinto-da-Rocha
