# Supplementary material for: ﻿Comparative cytogenetics among populations of two Bothriurus species (Scorpiones, Bothriuridae)
Source: Comp Cytogenet. 2025 Nov 13;19:189–208. doi: 10.3897/compcytogen.19.165160 (PMC12635637; doi:10.3897/compcytogen.19.165160)
Supplement: Supplementary material 1 — Supporting information [file comparative_cytogenetics-19-189_article-165160__-s001.docx]

**Supporting Information**

**Table S1.** List of specimens of *Bothriurus asper* and *Bothriurus rochai* used in this study including identification code and collection location of specimens. CE- Ceará, PB- Paraíba, PE- Pernambuco, PI-Piauí, RN- Rio Grande do Norte.

| **Specie** | **Voucher** | **State** | **Locality** |
| --- | --- | --- | --- |
| *Bothriurus asper* | CHNUFPI 10416 | PE | Igarassu |
| *Bothriurus asper* | CHNUFPI 10430 | PI | Teresina |
| *Bothriurus asper* | CHNUFPI 10431 | PI | Teresina |
| *Bothriurus asper* | CHNUFPI 10432 | PI | Teresina |
| *Bothriurus asper* | CHNUFPI 10433 | PI | Teresina |
| *Bothriurus rochai* | CHNUFPI 10401 | RN | Apodi |
| *Bothriurus rochai* | CHNUFPI 10402 | RN | Apodi |
| *Bothriurus rochai* | CHNUFPI 10403 | PI | Brasileira |
| *Bothriurus rochai* | CHNUFPI 10404 | PI | Brasileira |
| *Bothriurus rochai* | CHNUFPI 10405 | PI | Brasileira |
| *Bothriurus rochai* | CHNUFPI 10406 | PI | Brasileira |
| *Bothriurus rochai* | CHNUFPI 10407 | PI | Brasileira |
| *Bothriurus rochai* | CHNUFPI 10408 | PI | Brasileira |
| *Bothriurus rochai* | CHNUFPI 10409 | PI | Brasileira |
| *Bothriurus rochai* | CHNUFPI 10410 | PI | Piracuruca |
| *Bothriurus rochai* | CHNUFPI 10411 | PB | Cajazeiras |
| *Bothriurus rochai* | CHNUFPI 10412 | PB | Cajazeiras |
| *Bothriurus rochai* | CHNUFPI 10413 | PB | Cajazeiras |
| *Bothriurus rochai* | CHNUFPI 10414 | PI | Floriano |
| *Bothriurus rochai* | CHNUFPI 10415 | CE | Icó |
| *Bothriurus rochai* | CHNUFPI 10417 | PI | São Raimundo Nonato |
| *Bothriurus rochai* | CHNUFPI 10418 | PI | São Raimundo Nonato |
| *Bothriurus rochai* | CHNUFPI 10419 | PI | São Raimundo Nonato |
| *Bothriurus rochai* | CHNUFPI 10420 | PI | São Raimundo Nonato |
| *Bothriurus rochai* | CHNUFPI 10421 | PI | São Raimundo Nonato |
| *Bothriurus rochai* | CHNUFPI 10422 | PI | São Raimundo Nonato |
| *Bothriurus rochai* | CHNUFPI 10423 | PI | São Raimundo Nonato |
| *Bothriurus rochai* | CHNUFPI 10424 | PI | São Raimundo Nonato |
| *Bothriurus rochai* | CHNUFPI 10425 | PI | São Raimundo Nonato |
| *Bothriurus rochai* | CHNUFPI 10426 | RN | João Câmara |
| *Bothriurus rochai* | CHNUFPI 10427 | RN | João Câmara |
| *Bothriurus rochai* | CHNUFPI 10428 | PB | Maturéia |
| *Bothriurus rochai* | CHNUFPI 10429 | PB | Maturéia |
| *Bothriurus rochai* | CHNUFPI 10434 | CE | Quixadá |
| *Bothriurus rochai* | CHNUFPI 10435 | CE | Quixadá |
| *Bothriurus rochai* | CHNUFPI 10436 | CE | Quixadá |


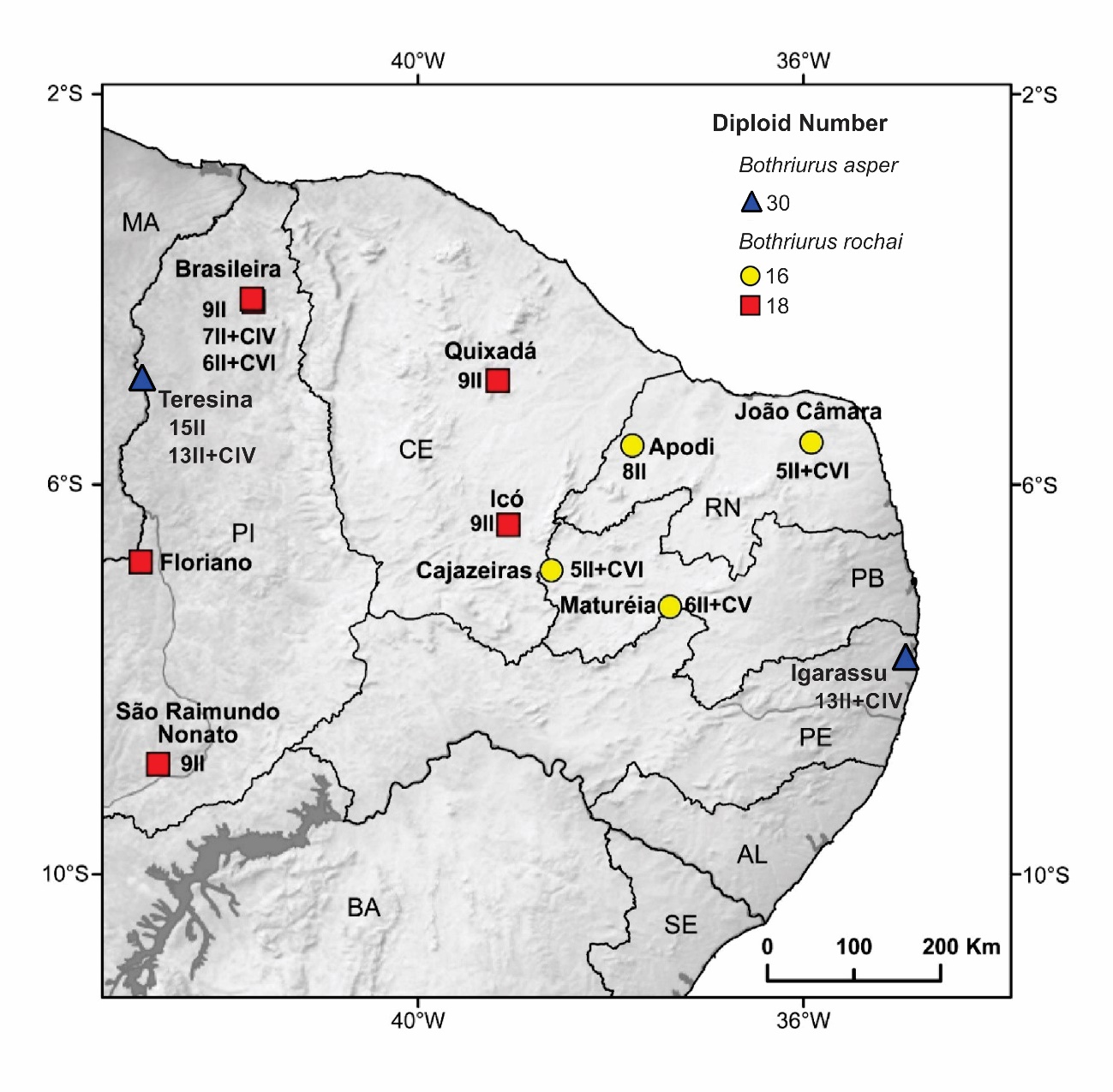


**Figure S1.** Map of the localities of all *Bothriurus asper* and *Bothriurus rochai* populations analyzed cytogenetically, indicating the chromosomal configurations observed in each case. II = bivalents; CIV = chain of four chromosomes; CV = chain of five chromosomes; CVI = chain of six chromosomes. *In the Floriano population, no post-pachytene cells were examined to determine the chromosomal configuration.

**
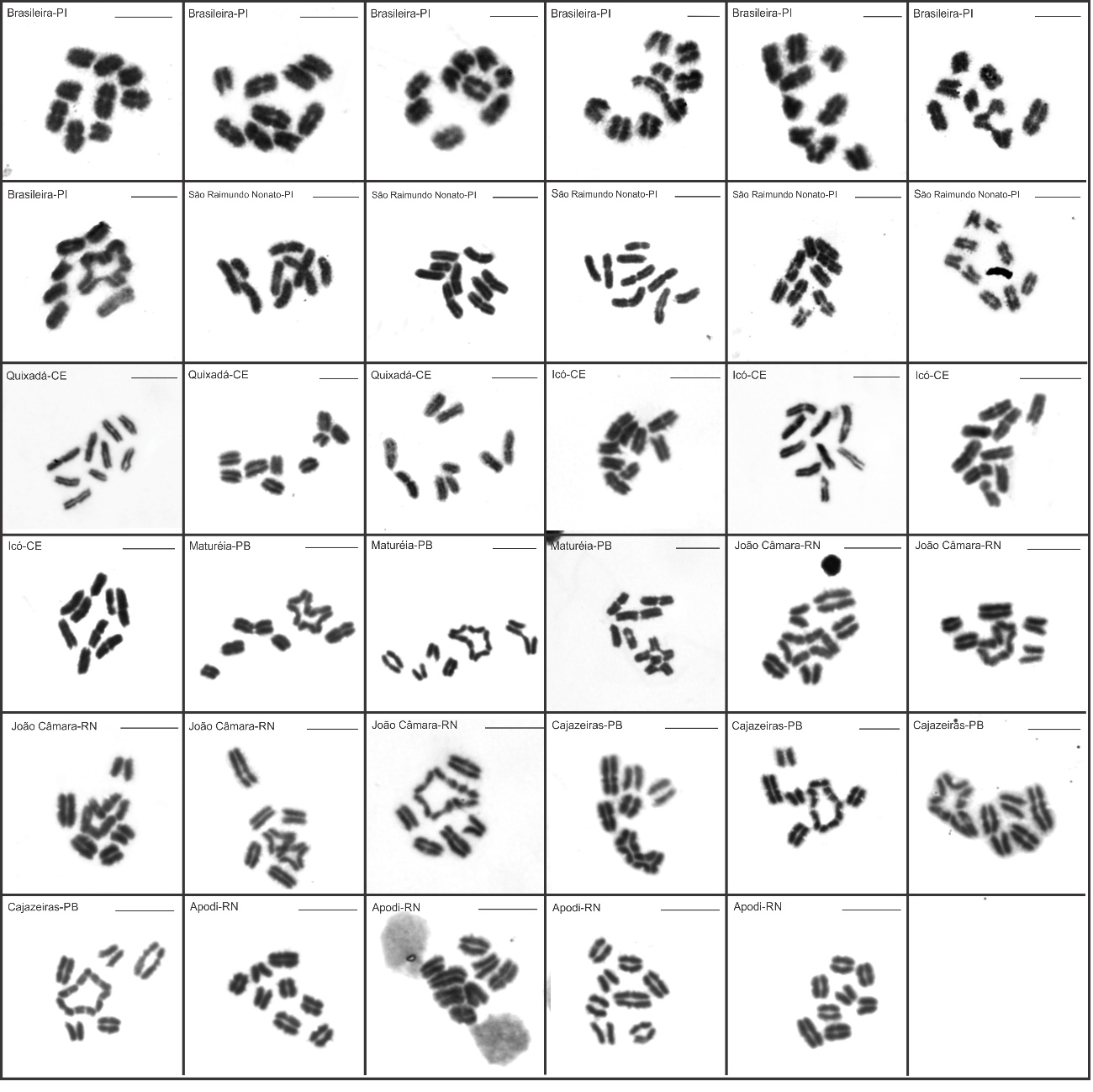
**

**Figure S2**. Post-pachytenic cells of the *B. rochai* populations that were measured
